# Factors Associated with the Development of Skin Lesions in Hospitalized Patients Admitted to a Nursing Preventive Care Program in Colombia

**DOI:** 10.17533/udea.iee.v43n3e04

**Published:** 2025-10-18

**Authors:** Gaby E. Escobar, Ángela F. Espinosa, Olga L. Cortés, Nicolás Molano-González

**Affiliations:** 1 Nurse, MSc. Researcher. Email: geescobarn@unal.edu.co. Principal researcher. https://orcid.org/0000-0001-9710-0398 Universidad del Rosario Colombia geescobarn@unal.edu.co; 3 Nurse, MSc. PhD Associate Researcher Email: ocortes@lacardio.org. https://orcid.org/0000-0003-0042-2935 Fundación Cardioinfantil Colombia ocortes@lacardio.org; 4 Statistician, MSc. Researcher and Professor. Email: Nicolas.molano@urosario.edu.co. https://orcid.org/0000-0002-9023-7479 Universidad del Rosario Colombia Nicolas.molano@urosario.edu.co; 5 Fundación Cardioinfantil - Instituto de Cardiología, La Cardio. Bogotá DC, Colombia. https://orcid.org/0000-0001-5458-8346 Fundación Cardioinfantil Fundación Cardioinfantil Instituto de Cardiología, La Cardio Bogotá DC Colombia; 6 Universidad del Rosario, Bogotá DC, Colombia Universidad del Rosario Universidad del Rosario Bogotá DC Colombia; 7 Group of Research on Public Health. School of Medicine and Health Sciences School of Medicine and Health Sciences Group of Research on Public Health School of Medicine and Health Sciences Colombia; 8 Clinical Research Group. School of Medicine and Health Sciences School of Medicine and Health Sciences Clinical Research Group School of Medicine and Health Sciences Colombia

**Keywords:** risk factors, preventive health services, pressure ulcer, dermatitis, nursing care, factores de riesgo, servicios preventivos de salud, úlcera por presión, dermatitis, cuidado de enfermería, fatores de risco, serviços de saúde preventivos, úlcera por pressão, dermatite, cuidados de enfermagem

## Abstract

**Objective.:**

This work sought to determine the factors associated with the development of skin lesions among patients hospitalized and admitted to a preventive skin care program carried out by nursing.

**Methods.:**

Analytical observational study of cases and controls, which included a sample of 150 cases and 300 controls hospitalized in a clinic with tier IV level of complexity in Bogotá (Colombia). A classification and regression tree was developed to explore the complex interactions that define cases and controls.

**Results.:**

According to the decision tree, the factors that represent greater probability for the development of skin lesions in the study population were the preventive use of hydrocolloid dressings, hospital stay > 12 days, BMI > 23, incontinence, diagnosis upon admission related with cardiovascular problems and peripheral vascular disease, cancer, surgery, or respiratory failure.

**Conclusion.:**

Development of skin lesions was related with the interaction of different clinical conditions presented by the patients. Integration of this knowledge is essential for structuring preventive care programs in high-complexity hospitals and in formulating individualized care plans.

## Resumen

**Objetivo.:**

Determinar los factores asociados con el desarrollo de lesiones cutáneas entre pacientes hospitalizados y admitidos en un programa de cuidado preventivo de la piel llevado a cabo por enfermería.

**Métodos.:**

Estudio observacional analítico de casos y controles. Se incluyó una muestra 150 casos y 300 controles hospitalizados en una clínica de cuarto nivel de complejidad de Bogotá (Colombia). Se construyó un árbol de clasificación y regresión para explorar interacciones complejas que definen casos y controles.

**Resultados.:**

De acuerdo con el árbol de decisiones, los factores que representan una mayor probabilidad para el desarrollo de lesiones en piel en la población del estudio fueron el uso de apósitos hidrocoloides de manera preventiva, la estancia hospitalaria superior a 12 días, IMC >23, incontinencia, diagnóstico de admisión relacionado con problemas cardiovasculares y enfermedad vascular periférica, cáncer, cirugía o falla respiratoria.

**Conclusión.:**

El desarrollo de lesiones cutáneas se relacionó con la interacción de diferentes condiciones clínicas presentadas por los pacientes. La integración de este conocimiento es esencial en la estructuración de programas de cuidado preventivo en hospitales de alto nivel y en la formulación de planes de cuidado individualizado.

## Resumo

**Objetivo.:**

Determinar os fatores associados ao desenvolvimento de lesões cutâneas em pacientes hospitalizados em um programa de cuidados preventivos de enfermagem com a pele.

**Métodos.:**

Trata-se de um estudo observacional, analítico, do tipo caso-controle. Foi incluída uma amostra de 150 casos e 300 controles hospitalizados em uma clínica de nível superior em Bogotá (Colômbia). Uma árvore de classificação e regressão foi construída para explorar interações complexas que definem casos e controles.

**Resultados.:**

De acordo com a árvore de decisão, os fatores que representaram maior probabilidade para o desenvolvimento de lesões cutâneas na população estudada foram o uso de curativos hidrocoloides para prevenção, internação hospitalar superior a 12 dias, IMC superior a 23, incontinência, diagnóstico de admissão relacionado a problemas cardiovasculares e doença vascular periférica, câncer, cirurgia ou insuficiência respiratória.

**Conclusão.:**

O desenvolvimento de lesões cutâneas esteve ligado à interação de diferentes condições clínicas apresentadas pelos pacientes. A integração desse conhecimento é essencial na estruturação de programas de cuidados preventivos em hospitais de alto nível e na formulação de planos de cuidados individualizados.

## Introduction

Among the skin lesions related with healthcare and occurring during the hospitalization of adult patients, there are skin disorders, such as incontinence-associated dermatitis (IAD), pressure injuries (PI), and medical adhesive related skin injuries (MARSI), among others.^1^ These lesions are considered a public health problem, given that they entail serious problems, threats and risks that include health complications, increased treatment, prolonged hospital stay, and repercussions on the quality of life of patients and caregivers.^2^ In addition, they generate economic repercussions and increase the work burden in healthcare teams, increasing costs for healthcare systems due to the need for special nursing care to heal these injuries, use of special pharmaceutical products, or need for surgery.^3^ Although most of these lesions are preventable, the evidence reported in some studies shows high prevalence,^2^^-^^7^ similar to that observed in the study institution where these three types of lesions (PI, IAD, and MARSI) were the most prevalent.

The European Pressure Ulcer Advisory Panel (EPUAP), the National Pressure Injury Advisory Panel (NPIAP), and the Pan-Pacific Pressure Injury Alliance for Pressure Lesions (PPPIA)^1^ define a PI as “a localized injury to the skin and/or underlying tissue, usually over a bony prominence, as a result of pressure, or pressure in combination with shearing forces”. Further, IADs are a type of skin lesion defined as “an inflammation and/or erosion of the skin associated with exposure to urine or feces”;^8^ and, finally, the medical adhesive related skin injuries (MARSI) defined in 2013 by McNichol *et al*.,^9^ as the onset of an “erythema and/or other manifestation of skin abnormality (which includes, among others, vesicles, blisters, erosion, or tearing), which persists during 30 minutes or more after removing the adhesive”.

Regarding PI, in the United States, 3% hospital prevalence is reported and 11% in intensive care units (ICUs).^2^ In Spain, data from the sixth national study on prevalence conducted by the National Group for the Study and Advisory of Pressure Ulcers and Chronic Injuries (GNEAUPP, for the term in Spanish) indicate increased prevalence with relation to the previous study due to the increase of patients with these types of lesions during the pandemic, prevalence in social-health residences and centers was 9.28%.^3^ In Colombia, observational studies high level of complexity hospitals reported 14% prevalence in internal medicine, emergency and ICU services in Bogotá,^4^ and 6% in Bucaramanga.^5^ In turn, IADs have a 4.3% prevalence in the United States and Canada, developing in 22% of patients with fecal incontinence, a key risk factor.^6^ In Norway, in 2018, IAD prevalence was reported around 16.5%.^7^ Lastly, MARSIs are the least studied lesions, with limitations in the evidence reported. An observational study in a hospital in the United States, conducted in medical-surgical ICUs and a cardiovascular telemetry unit, reported a daily prevalence between 3.4% and 25% (mean 13%) and a severity prevalence between 8 to 149 per 1000 product-days.^10^

Among the risk factors contributing to skin lesions acquired during hospitalization are advanced age, immobility, use of certain medications and the need for life support, among others, which compromises significantly the adult patient’s condition. These factors are compounded by the presence of comorbidities and the loss of functional capacity, which tend to be associated with more prolonged hospital stays.^2^ Likewise, patients in ICUs and in certain hospitalization areas tend to have a critical health status, with hemodynamic disorders and special pharmacological requirements. This situation, added to greater overall frailty, presence of pain, and limitation in mobility capacity increases significantly the risk of developing these types of lesions.^4^

To this series of factors we can add the fact that the preventive care strategies used in healthcare institutions, obtained from Clinical Practice Guides, have low and/or very low evidence levels, which increases uncertainty about the impact of certain interventions not proven effective (benefit or harm), which could turn them into an additional factor of possible risk or damage to the skin.^5^ Preventing these lesions would lead to improving the quality indicators of healthcare institutions and the quality of life of patients, families, and caregivers.^6^ This prevention requires a multidisciplinary approach that provides care comprehensively. Within this approach, nursing is responsible for identifying risk factors, through the use of risk assessment scales, and scales on leadership in implementing prevention strategies.^7^

The National Group for the Study and Advisory of Pressure Ulcers and Chronic Injuries recommends products for the care of healthy or fragile skin, such as hyper-oxygenated fatty acids, which favor cell regeneration, stimulate collagen formation, improve microcirculation, and protect against friction, although their effectiveness lacks sufficient scientific support.^10^ Zinc oxide creams and various dressings are also used, although these can make it difficult to see the skin, interfere with the adhesion of other products, and require specific agents for removal.^11^ Finally, moisturizers and emollients help retain water, protecting and hydrating the skin, making them effective in preventing lesions due to humidity and in extremely dry or damaged skins.^11^

Assessment of hospital quality has promoted the creation of strategies to prevent skin lesions during hospitalization. This study was carried out in a healthcare institution that has a preventive skin program, implemented by a cross-functional team, based on the implementation of evidence-based guides from the Registered Nurses Association of Ontario, focused on prevention, promotion, and safe treatment of patients’ skin to avoid adverse events. The program includes support surfaces, fatty acids, protective foam, position changes, and a clock in each room to program and notify of position changes. The program’s principal objective is to deepen, update, and disseminate to the institution’s nursing, medical, and paramedical staff the concepts required to guarantee the implementation of preventive actions that allow early identification of skin lesions, avoid their development, and promote the establishment of timely and adequate preventive treatment, thus, contributing to improving the quality of life and recovery of the health status of patients. 

Despite these strategies, the prevalence of skin lesions in the institution is still high, and not all the factors associated with their appearance in hospitalized patients are known with certainty. Due to this, an analytical, observational study was proposed to identify them. The three lesions with the highest clinical prevalence (PI, IAD, and MARSI) were evaluated, according to the level of complexity and dependence of patients treated by this institution, recognized as a national reference center.

The aim of this study was to identify factors linked with the development of pressure injuries, dermatitis associated with incontinence and medical adhesive related skin injuries, in hospitalized patients admitted to a nursing preventive care program.

## Methods

Design. Analytical observational study of cases and controls, carried out in a clinic with high complexity level of care in the city of Bogotá, Colombia.

Participants. The study used the clinical charts of patients admitted to the skin lesion preventive care program between January 2018 and December 2020. The inclusion criteria were patients aged ≥ 18 years, who received evaluation through consultation by the institutional nursing skin injury prevention group, and who were prescribed preventive care by this sane group. 

Sample size. Epi Info software version 7.2 of 2018 was used, employing a ratio of two controls per case, a confidence level of 95%, and a power of 90%. Based on the literature, being 70 years of age or older was considered a risk factor for pressure injuries, with the percentage of people > 70 years of age without this type of injury being 17% vs. 30.5% of people over this age with this type of injury (OR of 2.14).^10^ Thus, a minimum sample size was obtained of 147 cases and 293 controls, which was approximated to 150 and 300, respectively. The study included patients > 18 years of age who had been hospitalized in medical services, and in ICU and who had been evaluated and followed up by the Injury Prevention and Skin Care Group, considering as cases those patients who developed a PI, IAD, and MARSI type skin lesion acquired during hospitalization, and as controls those who did not develop a PI, IAD, and MARSI type skin lesion acquired during hospitalization. Random selection had been planned for both controls and cases. Once the database was reviewed, it was identified that of the 1,459 patients in the preventive program there were 150 cases, so it was decided to include all of them. With the controls, the selection was made randomly via the Excel program.

Variables. (1) Independent variables (i) demographic factors (age and sex); (ii) clinical factors (body mass index); (iii) history of comorbidities of cardiovascular, renal, and neurological origin, among others; (iv) diagnosis upon admission; (v) score according to the Braden scale at the first assessment of the skin care group; (vi) treatments administered during hospitalization (vasoactive medications, mechanical ventilation, antibiotics, sedatives, dialysis); (vii) presence of incontinence; (viii) skin lesion prevention treatments prescribed by this same group (use of lubricants, dressings, or fatty acids); (ix) length of hospital stay; (x) hospitalization service; (xi) type of nutrition and type of incontinence. (2) Dependent variables: Presence of any skin lesion (IAD, MARSI, and PI analyzed together). These lesions were diagnosed by the skin care and prevention group from the study institution. In addition, information was gathered about the characteristics of the lesions found.

Procedures. The revision and initial selection of patients was conducted from a database that included 2,225 patients evaluated in prevention consultation by the nursing group specialized in skin care, between January 2018 and December 2020. This database only included general information of the patients.

As a first step, the data were refined by eliminating patients < 18 years of age, reducing the total to 1,720 records. Subsequently, the types of skin lesions targeted by the study (PI, IAD, and MARSI) were identified, excluding 40 patients with other types of lesions, leaving 1,680 cases. Then, duplicate records corresponding to the same hospitalization period were eliminated, which excluded 221 patients and resulted in a final total of 1,459.

From this final group, 150 patients were identified with one of the three types of skin lesions studied, who were defined as the cases. For the controls, 300 patients were selected via random sampling from the same database, discounting the cases. 

Thereafter, the information in the database with respect to the study variables was complemented through the review of clinical records in the institution’s documentation system. Personal data, such as names and identity documents, were anonymized, assigning each patient a unique numerical code. All study information was compiled in a protected Excel file, designed to allow systematic and organized data collection.

Ethical considerations. The study was approved by the Research Ethics Committee (CEIC) IRB00007736 [ID 11-2021]. 

Data analysis. The different types of skin lesions (PI, IAD, and MARSI) were analyzed together. In the univariate analysis, qualitative variables are reported with absolute and relative frequencies, while quantitative variables are reported with medians and interquartile ranges. For the description of the cases and controls, the same measures mentioned are reported along with their corresponding measure of effect ( mean difference or Odds Ratio) accompanied by their 95% confidence interval and their p-*value*, according to the Chi squared or Mann Whitney U tests. For polytomous variables, The reference categories were chosen because they corresponded to the lowest risk or, when they could not be classified as lower or higher risk, the category with the lowest prevalence. To better understand the characteristics associated with the cases and controls, a classification and regression tree was constructed,^11^ which was used to find automatically complex interactions among covariables of clinical interest. As outcome of this analysis, groups or profiles of patients were identified that share clinical characteristics with differential distribution of skin lesions. The variables included in the decision tree were sociodemographic variables, clinical variables, and treatment variables, which made up 29 variables. All statistical analyses were performed in R software version 4.3.2.

Validity, reliability, and rigor. The initial database was carried out by nursing professionals trained in prevention of skin lesions and with basic knowledge of Excel. This database only included general information of the patients. During the data collection (2019-2020), The database was filled out by the principal researcher with the information recorded in the patient's medical history. The principal researcher, along with a coresearcher, controlled and validated the data recorded in the database, verifying the completeness of the information and identifying inconsistent data that required clarification with a new review of the medical history. Thereafter, the veracity of the information recorded in the database was randomly compared with that recorded in the medical history.

## Results

The study population was comprised by 450 patients, of which 150 were cases and 300 were controls, where it was found that the minimum age was 18 years and the maximum was 107 years, with a median of 67. The median in the group of cases was lower with respect to the controls 66 and 68, respectively (Table 1); 58% (*n* = 259) of the population were men, with higher percentage in the cases than in the controls. Skin lesion prevalence in the preventive program was 10.21% (150/1469).

Statistically significant differences were observed in the population study in variables of weight, hospital stay and number of follow-ups, finding higher medians in each of the variables for the group of cases (Table 1). 

**Table 1:** Quantitative characteristics of the study population

Variables	Total (*N* = 450) Me [Mín-Max]	Cases (*n* = 150; 33.3%) Me [Mín-Max]	Controls (*n* = 300; 66.7%) Me [Mín-Max]	***p*-value**
Age (years)	67 [18- 107]	66 [19- 107]	68 [18- 100]	0.32
**Weight *(Kg)***	65 [ 23- 130]	68 [32- 130]	63.7 [23-130]	0.04
**Height *(m)***	1.63 [ 1.39-1.86]	1.65 [1.40- 1.85]	1.61 [ 1.39- 1.86]	0.15
Body mass index	24.03 [10.96-47.17]	24.68 [13.49- 44]	23.87 [10.96- 47.17]	0.15
Days of hospital stay	19 [1- 270]	28 [ 2- 270]	14.50 [1-140]	≤0.001
Number of follow ups	1 [ 1-14]	2 [ 1- 8]	1 [ 1-14]	≤0.001

*BMI: body mass index

The bivariate analysis identified significant differences for developing skin lesions between cases and controls in the variables of history of hypertension and peripheral vascular disease; being admitted to any hospitalization service (not ICU), having a high-risk assessment for pressure injuries, according to Braden’s scale; being exposed to management with mechanical ventilation, treatment with sedatives, antibiotics, vasoactive substances, and with prescription of parenteral nutrition. Likewise, these patients had greater incontinence and received preventive care with hydrocolloid dressings and with fatty acids. 

Neurological disorder, as diagnosis upon admission, was identified with greater prevalence in the control group (Table 2).

**Table 2:** Qualitative characteristics of the study population

Variables	Cases (*n* = 150) *n* (%)	Controls (*n* = 300) *n* (%)	*p-value*	OR (95% CI OR)
Sex				
Male	100 (66.67)	159 (53.00)	<0.001	1.77 (1.18-2.68)
Female	50 (33.33)	141 (47.00)		
Comorbidities				
Hypertension	56 (37.3)	165 (55.0)	<0.001	0.48 (0.32 - 0.72)
Peripheral Vascular Disease	35 (23.3)	48 (16.0)	0.05	1.59 (0.98 - 2.60)
Cerebrovascular disease	12 (8.0)	23 (7.7)	0.90	1.04 (0.50 - 2.16)
Dementia	4 (2.7)	20 (6.7)	0.08	0.39 (0.12 - 1.14)
Neurological disorder	15 (10.0)	71 (23.7)	<0.001	0.36 (0.19 - 0.65)
Respiratory disease	40 (26.7)	74 (24.7)	0.64	1.11 (0.71 - 1.73)
Diabetes	36 (24.0)	61 (20.3)	0.37	1.23 (0.77 - 1.97)
Incontinence	75 (50.0)	83 (27.7)	<0.001	2.60 (1.73 - 3.93)
Urinary	1 (1.3)	5 (6.0)	0.21	2.34 (0.555 -21.7)
Urinary and/or fecal	74 (98.7)	78 (94.0)		
Therapeutic				
Mechanical ventilation	72 (48.0)	105 (35.0)	<0.001	1.71 (1.15 - 2.55)
Dialysis	7 (4.7)	18 (6.0)	0.56	0.77 (0.29 - 1.84)
Antibiotic	105 (70.0)	174 (58.0)	0.01	1.68 (1.11 - 2.57)
Sedative	78 (52.0)	108 (36.0)	<0.001	1.92 (1.29 - 2.86)
Vasoactive	60 (40.0)	87 (29.0)	0.01	1.63 (1.08 - 2.46)
Prevention				
Use of Hydrocolloid dressing	54 (36.0)	23 (7.7)	<0.001	6.71 (3.95 - 11.74)
Zinc oxide	103 (68.7)	220 (73.3)	0.29	0.79 (0.51 - 1.23)
Hospitalization service				
Intensive care	104 (69.33)	161 (53.67)		
Hospitalization	46 (30.67)	139 (46.33)	<0.001	0.51 (0.33 - 0.77)
Diagnosis upon admission				
Cancer *	11 (7.3)	7 (2.3)		
Respiratory failure	49 (32.7)	69 (23.1)	0.12	0.45 (0.16 - 1.24)
Cardiovascular disorder	41 (27.3)	67 (22.4)	0.07	0.38 (0.14 - 1.08)
Programmed and/or emergency surgery	20 (13.3)	45 (15.1)	0.02	0.28 (0.09 - 0.83)
Neurological disorder	8 (5.3)	55 (18.4)	<0.001	0.09 (0.02 - 0.30)
Sepsis/infection	21 (14.0)	35 (11.7)	0.08	0.38 (0.12 - 1.13)
Type of nutrition				
Oral*	37 (24.7)	157 (52.3)		
Enteral	52 (34.7)	112 (37.3)	0.01	1.97 (1.21 - 3.20)
Parenteral	54 (36)	27 (9.0)	<0.001	8.48 (4.73 - 15.22)
Mixed	7 (4.7)	4 (1.3)	<0.001	7.42 (2.06 -26.69)
Braden’s scale				
Low Risk*	2 (1.3)	10 (3.3)		
Moderate Risk	9 (6.0)	49 (16.3)	0.92	0.91 (0.17 - 4.91)
High Risk	75 (50)	179 (59.7)	0.34	2.09 (0.44 - 9.79)
Very High Risk	64 (42.7)	62 (20.7)	0.03	5.16 (1.08 - 24.50)
Type of Lubricant				
Fatty acids	138 (92.0)	210 (70.0)		
Other lubricants	3 (2.0)	42 (14.0)	<0.001	0.10 (0.03 - 0.35)
None	9 (6.0)	48 (16.0)	<0.001	0.28 (0.13 - 0.60)

### Multivariate analysis

**Figure 1:**
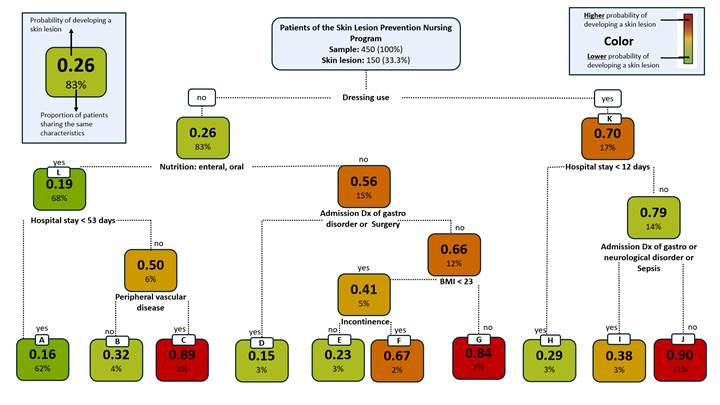
Diagram of the classification and regression tree

Figure 1 shows the diagram of the classification and regression tree. Of the 29 variables included, the classification and regression tree algorithm - automatically and objectively based on the data - determined what are the relevant variables that construct the tree. This tree shows 10 groups or clinical profiles (identified with letters A to J) based on seven variables that proved having relevance in developing skin lesions. These variables were: use of hydrocolloid dressings, type of nutrition, duration of the hospital stay, diagnosis upon admission, peripheral vascular disease, body mass index, and presence of urinary and/or fecal incontinence (Figure 1). It is also important to highlight that the use of hydrocolloid dressings and the hospital stay were the variables with greater importance when constructing the decision tree. 

Patients characterized between profile A and profile G, who did not receive care with hydrocolloid dressings, had a lower probability of developing a skin lesion than patients characterized and included from profiles H to J, who were characterized for having received care with hydrocolloid dressings [26% Vs. 70%], respectively (Figure 1).

Profiles A, B, D, E, H, identified in the decision tree analysis, had a low probability of developing skin lesions (16%, 32%, 15%, 23%, and 29%), respectively. Profile I had an intermediate 38% probability; and profiles C, F, G, and J had a higher probability of developing a skin lesion (89%, 67%, 84%, 90%), respectively (Figure 1).

Among the group of the A, B, D, E, and H profiles (Figure 1, green tones), profile A gathered the higher number of patients from the sample (279/450 patients, 62%), which had a 16% probability of developing a skin lesion. These patients were characterized for not using hydrocolloid dressings, receiving enteral or oral nutrition, and for having a hospital stay < 53 days. Profile B, characterized for including patients who did not use hydrocolloid dressings, with hospital stay > 53 days, and without peripheral vascular disease, had 32% probability of developing skin lesions. Profile D included patients characterized for not using hydrocolloid dressings, being admitted due to gastrointestinal problems or admission to surgery, had 15% probability. Profile E, characterized for not using hydrocolloid dressings, for including patients with non-surgical diagnosis, with BMI < 23, and without incontinence, had 23% probability. Profile H, characterized for including patients with hospital stay <12 days, but protected with hydrocolloid dressings had 29% probability.

In the group of profiles C-F-G-J (Figure 1, red and orange), 22% of the sample was identified and a higher probability of skin lesion (99/450 patients). Profiles C, F, and G concentrated 11% of patients with higher proportion of skin lesion. Profiles F and G shared the following characteristics: lack of use of hydrocolloid dressings, nutrition other than enteral or oral, and diagnosis other than surgery or gastrointestinal disorder. Patients included in profile C (2% of the sample), who were characterized for not using hydrocolloid dressings, received enteral/oral nutrition, had a hospital stay > 53 days, and had an additional factor of peripheral vascular disease showed a high probability of 0.89 of developing a skin lesion. Patients included in profile G (7% of the patients) with an additional factor of BMI > 23 had 84% probability of skin lesion. Patients included in profile F (2% of the sample) with BMI < 23, but with an additional factor of urinary incontinence had 67% probability of developing a skin lesion. Profile J, which characterized patients identified with cancer diagnosis, respiratory failure, and cardiovascular disorder on admission, with hospital stay > 12 days, and who received care with hydrocolloid dressings, corresponded to 11% of the patients [50/450], had a high (90%) probability of developing a lesion.

Patients who shared the same characteristics, except not having peripheral vascular disease (profile B) had nearly three times less likelihood of a skin lesion than those who did have peripheral vascular disease (89% profile C Vs. 32% profile B) (OR = 14.14; 95% CI = 1.91-413.31; *p*-value = 0.01275).

With respect to diagnosis upon admission, among the patients who shared the same profile with the only difference in this variable, the difference in the proportion decreased from 90% to 38% (profile J Vs. I). The proportion of skin lesion was greater in those patients whose diagnosis upon admission included cancer, respiratory failure, and cardiovascular disorder compared with those whose diagnosis was surgery for gastrointestinal disorder, neurological disorder, or sepsis (OR = 13.23; 95% CI = 3.20-63.89; *p*-value = 0.0003).

In patients sharing the characteristics of not using hydrocolloid dressings, parenteral nutrition, BMI < 23, diagnosis upon admission corresponding to neurological disorder, cardiovascular disorder, sepsis, cancer, and respiratory failure, except for having or not having urinary incontinence, the proportion of skin lesion changes from 67% in profile F Vs. 23% in profile E (OR = 5.88; 95% CI = 0.93-48.76; *p*-value = 0.0789). Profile D has 3% of the population corresponding to 14 patients; of those, 15% developed skin lesion (2 patients); this profile is characterized for not using hydrocolloid dressings, parenteral nutrition, and diagnosis upon admission of gastrointestinal disorder and surgery. Finally, profile H also had 3% of the population and 29% of them (4 patients) had skin lesion. This profile gathered individuals who used hydrocolloid dressings and had a hospital stay < 12 days.

## Discussion

This study contributes to progress in identifying determining factors in the onset of PI, IAD, and MARSI acquired in patients hospitalized in a high-complexity center. The decision tree permitted finding complex interactions that could not have been discovered with classic methods, such as logistic regression. The greatest proportion of skin lesions was found in patients who used hydrocolloid dressings, had a hospital stay > 12 days, with BMI > 23, incontinence, and diagnosis upon admission related with cardiovascular problems and peripheral vascular disease, cancer, surgery, or respiratory failure. 

Profile J is striking given that hydrocolloid dressings in the program evaluated has a preventive indication, which may indicate that in individuals with diagnosis upon admission of surgery, cancer, cardiovascular disorder, and respiratory failure who have been hospitalized for over 12 days, the hydrocolloid dressing would not be fulfilling its function. Evidence about the preventive and therapeutic effectiveness of using dressings is controversial,^12^ and depends on the type of dressing.^5^^,^^13^ Moreover, patients admitted to hospitalization due to the already mentioned diagnoses and with prolonged hospital stay, with prolonged bed rest have high fragility that favors developing skin lesions and, hence, require greater care.

Regarding profile C, patients with peripheral vascular disease as comorbidity, with a hospital stay > 53 days, enteral or oral nutrition, and who did not use hydrocolloid dressings had 89% probability of skin lesion, with the chance of developing skin lesion 13 times higher compared with patients from a similar profile, except for peripheral vascular disease. This comorbidity is based on vascular stenosis,^14^ which can limit tissue nutrition, and added to a prolonged stay can increase the risk of developing a skin lesion.

Patients who did not use hydrocolloid dressing, who were being fed via parenteral and mixed nutrition with diagnosis upon admission of cancer, respiratory failure, and cardiovascular disorder and with BMI > 23 had 84% probability of skin lesion (profile G), with 12 times greater chance of developing the outcome compared to patients with the same profile, but with a different diagnosis. With respect to this finding, regarding respiratory failure, it is worth noting that the last year of the study corresponded to the COVID-19 pandemic, therefore, the complexity in managing these patients led to changing different institutional factors that could have affected the presence of lesions during this year. Vowden *et al*.,^15^ claim that during the first wave of COVID-19, caregivers focused on treating the acute disease, which is why preventing skin lesions was not a priority. The impact of COVID-19 on training, the workforce, and the infrastructure, with a redistribution of beds and expansion of ICU facilities led to a notable increase of the risk. For patients with cancer or with any cardiovascular disorder, the disease’s specific conditions can affect tissue nutrition.^16^ Additionally, mixed or parenteral nutrition may indicate a more complex disease state that increases the patient’s vulnerability to developing skin lesions.

Hospital stay was a factor differentially associated with skin lesions, but always important (profile L and K). The risk of developing lesions increases in direct proportion to the length of hospital stay, the patient's aggravated condition makes movement impossible and leads to prolonged exposure to friction, pressure and shear, ultimately leading to the development of a lesion.^7^ In a study conducted in Iran in patients with > 10 days of stay in the ICU, the risk of developing skin lesions was four times greater with respect to patients with less days of hospitalization.^17^ Parenteral, enteral, and mixed nutrition variables behaved as a risk factor; this could be explained because these patients have more serious health conditions (ICU stay, mechanical ventilation, sedation, among others) that would lead to increasing the risk of acquiring skin lesions. According to the literature, direct relationship exists between malnutrition and development of skin lesions, as protection over bony prominences is reduced due to loss of muscle tissue and fat.^18^ In addition, inflammation produced with skin lesions demands increased nutritional needs.^19^

Long-term fecal and/or urinary incontinence cause a humid environment and maceration that alter the integrity of the skin and increase the risk of developing skin lesions. A literature review by Emilia *et al*., in 2019 identified that the highest prevalence of pressure injury was found in patients with urinary and/or fecal incontinence because humidity increases friction and diminishes the skin’s resistance to existing loads.^20^^) I^n the findings herein, the chance of developing PI was nearly five times more in patients sharing similar characteristics, except urinary incontinence.

Strengths and limitations. The study identified factors associated with the development of skin lesions when patients are in a nursing care preventive program. A strength of this research was the power of the sample size to assess factors associated with developing skin lesions in the study population. Furthermore, the lesions were identified by nurses with expertise in skin care. Among the limitations, it must be stated that the study population included patients selected specifically in a high-complexity clinic and due to this the findings are only comparable with patients from similar institutions. Also, the analysis of the three types of lesions (PI, IAD, and MARSI) together can be a limitation, since it generalizes the results and cannot provide specific preventive measures for each type of lesion. Because this is a study with retrospective source, bias is possible in reporting data in the medical history; however, the institution has information quality control systems, given that it is supervised by national and international entities for its high-quality accreditation. The sociodemographic and hospitalization characteristics that could have been a potential source of confusion were controlled through the multivariate analysis with the decision tree.

Conclusion. The factors that represent greater probability of developing PI, IAD, and MARSI type skin lesions in hospitalized adult patients were the preventive use of hydrocolloid dressings, prolonged hospital stay > 12 days, diagnosis upon admission of cardiovascular and vascular origin, BMI > 23, and urinary incontinence. The nurses and health team must comprehensively assess patients, bearing in mind that there is no unique clinical characteristic for developing a skin lesion; it takes place in function of different factors that when interacting with each other increase or diminish the possibility of occurrence.

Contributions by the authors. Gaby Escobar, Angela Espinosa-Aranzales, Olga Cortés, and Nicolas Molano made substantial contributions to the conception and design, or data acquisition, or data analysis and interpretation. All the authors participated in drafting the manuscript or in the critical revision of the important intellectual content. All the authors approved the final version for publication. Every author had to have participated sufficiently in the work to assume public responsibility for the appropriated parts of the content. All the authors agreed to be responsible for all the aspects of the work to guarantee that questions related with the accuracy or integrity of any part of the work are investigated and resolved adequately.
